# A protocol for a systematic review of economic evaluation studies conducted on neonatal systemic infections in South Asia

**DOI:** 10.1186/s13643-017-0648-7

**Published:** 2017-12-12

**Authors:** Shruti Murthy, Denny John, Isadora Perpetual Godinho, Myron Anthony Godinho, Vasudeva Guddattu, N. Sreekumaran Nair

**Affiliations:** 1 Department of Statistics, Prasanna School of Public Health, Manipal Academy of Higher Education, Level 6, Health Science Library Building, Madhav Nagar, Manipal, Karnataka 576104 India; 2 The Campbell Collaboration, New Delhi, India; 3 Department of Health Services Research, CAPHRI Care and Public Health Research Institute, Maastricht University, Maastricht, The Netherlands; 4 School of Economics, University of Nottingham (Malaysia campus), Semenyih, Malaysia; 5 Public Health Evidence South Asia (PHESA), Prasanna School of Public Health, Manipal Academy of Higher Education, Manipal, India; 6 Department of Biostatistics (Biometrics), Jawaharlal Institute of Postgraduate Medical Education and Research (JIPMER), Puducherry, India

**Keywords:** Systematic review, Protocol, Neonate, Systemic infections, Sepsis, Economic evaluation, South Asia

## Abstract

**Background:**

Neonatal systemic infections and their consequent impairments give rise to long-lasting health, economic and social effects on the neonate, the family and the nation. Considering the dearth of consolidated economic evidence in this important area, this systematic review aims to critically appraise and consolidate the evidence on economic evaluations of management of neonatal systemic infections in South Asia.

**Methods:**

Full and partial economic evaluations, published in English, associated with the management of neonatal systemic infections in South Asia will be included. Any intervention related to management of neonatal systemic infections will be eligible for inclusion. Comparison can include a placebo or alternative standard of care. Interventions without any comparators will also be eligible for inclusion. Outcomes of this review will include measures related to resource use, costs and cost-effectiveness. Electronic searches will be conducted on PubMed, CINAHL, MEDLINE (Ovid), EMBASE, Web of Science, EconLit, the Centre for Reviews and Dissemination Library (CRD) Database, Popline, IndMed, MedKnow, IMSEAR, the Cost Effectiveness Analysis (CEA) Registry and Pediatric Economic Database Evaluation (PEDE). Conference proceedings and grey literature will be searched in addition to performing back referencing of bibliographies of included studies. Two authors will independently screen studies (in title, abstract and full-text stages), extract data and assess risk of bias. A narrative summary and tables will be used to summarize the characteristics and results of included studies.

**Discussion:**

Neonatal systemic infections can have significant economic repercussions on the families, health care providers and, cumulatively, the nation. Pediatric economic evaluations have focused on the under-five age group, and published consolidated economic evidence for neonates is missing in the developing world context. To the best of our knowledge, this is the first review of economic evidence on neonatal systemic infections in the South Asian context. Further, this protocol provides an underst anding of the methods used to design and evaluate economic evidence for methodological quality, transparency and focus on health equity. This review will also highlight existing gaps in research and identify scope for further research.

**Systematic review registration:**

PROSPERO CRD42017047275

**Electronic supplementary material:**

The online version of this article (10.1186/s13643-017-0648-7) contains supplementary material, which is available to authorized users.

## Background

India, constituting about one-third of the world’s neonatal deaths, is the largest contributor to global neonatal mortalities [1]. The annual average rate of reduction of neonatal mortality has been slower compared to that of the infant and under-five mortality, thus increasing the fraction of neonatal mortality in relation to infant and under-five deaths [2]. Systemic infections, such as septicaemia, pneumonia, meningitis, osteomyelitis and arthritis, and urinary tract infections are responsible for more deaths than any other infectious disease among newborns and are surpassed only by prematurity and birth asphyxia [3, 4]. Among the systemic infections, neonatal septicaemia or sepsis, pneumonia and meningitis are responsible for about 25% of all newborn deaths [5, 6].

Neonatal systemic infections and their consequent impairments can lead to long-lasting health, economic and social effects on the neonate, the family and, cumulatively, the nation [7]. Numerous studies from Pakistan, India, Kenya and Vietnam on under-five children suggest that the direct and indirect costs of sepsis impose a significant economic burden upon the family, society and health system [8–11]. Economic evidence is increasingly recognized as an important input for decisionmaking to analyse impacts of changing patterns of resource allocation [12–14]. However, there is a lack of consolidated economic evidence for neonatal systemic infections from the developing world. Hence, this systematic review is being conducted to critically appraise and consolidate literature on economic evaluations of management of systemic infections among neonates in South Asia.

## Methods

This protocol has been developed based on “The Preferred Reporting Items for Systematic Reviews and Meta-Analyses Protocols” (PRISMA-P) [15] and registered in PROSPERO (CRD42017047275) [16]. The completed PRISMA-P checklist for our protocol is included as an additional file [see Additional file 1]. The systematic review will be conducted and reported in accordance with the PRISMA guidelines [17], the Cochrane Collaboration [18] and the National Institute for Health and Care Excellence (NICE) [19].

### Operational definitions

Neonatal systemic infections: Defined as including septicaemia, pneumonia, meningitis, osteomyelitis, arthritis and urinary tract infections among neonates [3].

South Asian regions: The list of regions included as “South Asian Association for Regional Cooperation (SAARC)” member countries will be used for our review. The member countries include Afghanistan, Bangladesh, Bhutan, India, Maldives, Nepal, Pakistan and Sri Lanka [20].

Full and partial economic evaluations: These will be defined and classified in accordance with the definitions provided by the Cochrane Campbell Economic Methods Group (CCEMG) [21].

### Criteria for considering studies for this review

#### Type of studies

##### Inclusion criteria

Economic evaluation studies in English will be included. It is unlikely that languages other than English would be the primary language in which the economic evidence reports would be published in South Asia. One of the authors (DJ) will help source potential studies in local languages through his networks at Health Economics Associations in Nepal, Bangladesh and Sri Lanka. Both trial-based and model-based economic evaluations will be eligible for inclusion.

The types of studies [21–23] that will be eligible for inclusion are:

1. Full economic evaluation: Cost-Effectiveness Analysis (CEA), Cost-Benefit Analysis (CBA), Cost-Utility Analysis (CUA) and Cost-Minimization Analysis (CMA)
2. Partial economic evaluations: Cost comparison/cost analysis, Cost outcome descriptions, Cost descriptions and Cost-of-illness (CoI) studies

##### Exclusion criteria

The following types of studies will be excluded:

1. Economic evaluations of neonatal systemic infections secondary to a disease/condition (e.g. surgery)
2. Studies reporting only quality of life (QoL), patient-reported outcomes and utilities without the costs
3. Reviews, editorials, commentaries or methodological articles. Bibliographies of systematic reviews will be utilized to examine relevant studies for inclusion. However, the reviews will not be eligible for inclusion.

#### Type of population

The population of interest will be neonates diagnosed with one or more systemic infections from any of the specified South Asian regions.

#### Type of intervention

Any intervention related to infection management (e.g. medical devices, guidelines, etc.) will be eligible for inclusion irrespective of the study settings (primary/secondary/tertiary health facilities and community). Comparison(s) can include either a placebo or an alternative standard of care or a do-nothing scenario.

#### Type of outcome measures [21]

Outcomes will include measures related to (A) resource use, (B) costs and (C) cost-effectiveness associated with the management of neonatal systemic infections.

(A) *Measures of resource use*: The number of units of resources used reported will be recorded. Measures of resource use will include specific resource items related to medical and non-medical categories. Examples of each have been provided below:
  (i) Medical: number and length (where available) of outpatient visits, consultations, hospitalizations (in-patient visits), provider services (physician, specialist, nursing, respiratory therapist, counsellor), drugs, supplies, devices and medical assistive equipment, investigations and procedures (clinical, laboratory, imaging).
  (ii) Non-medical: Number and/or duration (where available) of services (e.g. administration and housekeeping), overheads, equipment, transportation, meals, accommodation, relocation and moving, clothing, property losses, informal care (e.g. travel and waiting time), etc.
  (iii) Indirect: Number of hours/days of work lost (by the guardian/parent) as a result of the neonate’s illness
(B) *Measures of costs*: Cost outcomes will be reported in terms of
  (i) Cost estimates per specific resource item,
  (ii) Level of cost
  (iii) Category of cost

Measures of cost will include average cost estimates of specific resource items classified as direct medical, non-medical and indirect cost categories. The average total direct medical and direct non-medical costs will be reported, where available. The outcome measures will be captured as reported in the included studies to avoid subjective interpretation.

(C) *Measures of cost-effectiveness*: Measure of cost-effectiveness will be reported in “Incremental Cost-Effectiveness Ratio” (ICER), “Incremental cost per Quality-Adjusted Life Year (QALY)” “Incremental cost per Disability-Adjusted Life Year (DALY)”, “Incremental cost-benefit ratio” and net costs [21].

An additional file shows the table of planned outcomes of the systematic review [see Additional file 2].

### Search methods for identification of studies

#### Data sources

Electronic searches will be conducted on PubMed, CINAHL (EBSCO), MEDLINE (Ovid), Embase, Web of Science, EconLit, NHS Economic Evaluation Database (NHSEED) and NHS Health Technology Assessment (NHS HTA) (via The Centre for Reviews and Dissemination Library- CRD), Popline, the Cost Effectiveness Analysis (CEA) Registry, Pediatric Economic Database Evaluation (PEDE) and regional databases such as IndMed (indmed.nic.in), MedKnow (www.medknow.com) and Index Medicus for the South-East Asian Region (IMSEAR). No date restrictions will be applied in order to maximize the currency of the results. Conference proceedings will be searched on the websites of Health Technology Assessment International (HTAi), International Society of Pharmacoeconomics and Outcomes Research (ISPOR), International Health Economics Association (iHEA) and Guidelines International Network (GIN). Grey literature sources will include Research Papers in Economics (RePEc) and IDEAS. Back-referencing of bibliographies of included studies will be performed to examine the lists for potentially relevant studies [24].

#### The search strategy

A sensitive search strategy will be developed in accordance with the recommendations provided in the “Centre for Reviews and Dissemination’s (CRD) Guidance” for undertaking reviews in healthcare [25], search filters for various databases as recommended by the InterTASC Information Specialists SubGroup (ISSG) [26], after reviewing relevant literature of health economic reviews [24, 27, 28], and in consultation with subject experts and information scientists. The search strategy will include use of a combination of free text, indexing terms, database-specific limits and database-specific subject headings/vocabulary (e.g. MeSH). The first step will involve developing multiple search terms for each of the three domains, i.e. neonates (population), systemic infections (condition) and cost (outcome). Next, these search terms will be combined using “OR” within each domain. The domains will then be combined using “AND”. Additionally, adjacency operators will be used to limit the retrieval of irrelevant records. As a final step, database-specific filters will be used to limit the search to “Humans”, “English” and “South Asia”. If a database-specific filter is unavailable for limiting studies to “Human” population, the Boolean operator “NOT” will be used to remove animal studies as mentioned in the additional file. If a filter for the geographical location is unavailable to limit to South Asia, it will be included as the fourth domain in our search terms and will be combined using “AND” with the domains listed above. An additional file shows the preliminary search strategy for MEDLINE (Ovid) [see Additional file 2].

### Data collection and management

EndNote x7 will be used to manage search results and remove duplicate records. Study selection and data extraction will be performed in Microsoft Excel.

#### Study selection

The studies will be examined against pretested eligibility criteria by two authors (SM and IG) independently in three stages of title, abstract and full-text screening. Title and abstract study selection stages will be done to exclude obviously irrelevant reports. During title screening (first stage), titles rejected by both the authors will not be included in the review. Titles that are approved by either of the authors will be included for abstract screening (second stage). Abstracts accepted by either of the authors, in the second stage, will be included for full-text screening (third stage). Full-text records that are accepted by both the authors will be included in our review. Disagreements regarding the inclusion of full-text records will be resolved by discussion and reaching consensus in the presence of a third author (DJ). None of the authors involved in examining the relevance of studies will be blind to study details. The selected studies will subsequently be classified according to the list provided in the “Type of studies” section above [21–23].

#### Data extraction

Data extraction will be performed on a pilot-tested standardized form on Microsoft Excel by two authors (SM and IG) independently and reviewed by a third author (MG). The form will be structured based on the format and guidelines used to produce structured abstracts of economic evaluations for inclusion in the NHS Economic Evaluation Database (NHS EED) [29], the CCEMG [21], the “Consolidated Health Economics Evaluation Reporting Standards (CHEERS)” statement [30], and data items included in published studies [22, 31]. Disagreements between the authors will be resolved by discussion and consensus in the presence of a senior reviewer (DJ). Disagreement(s) that remain unresolved, despite attempts at reaching consensus, will be reported and discussed in our final review. An additional file shows the items for data extraction in detail [see Additional file 2].

#### Risk of bias assessment (RoB)

Risk of bias/methodological quality will be assessed independently by two authors (SM and MG) and reviewed by a third author (DJ) using the following tools:

- Trial-based economic evaluation studies: Cochrane Collaboration’s tool for assessing risk of bias [18], CHEERS statement [30], Consensus Health Economic Criteria (CHEC) Criteria list [32] and Drummond Checklist [33]
- Model-based economic evaluation studies: CHEERS statement [30], NICE guidelines [34] and Phillips checklist [35]
- Cost-Benefit Analysis studies: Benefit-Cost Validity scale [36]
- Cost-of-illness studies: Based on adapted checklist by Larg and Moss [37, 38]

Additionally, to evaluate the “transparency” of the cost estimates in included studies, the criteria developed by Fukuda and Immanaka will be used to classify studies into “transparency levels” depending on the clarity and the level of reporting of the cost components, quantity and unit price of resources and an estimate of each cost component [39]. The outcome of this appraisal and its implications will be discussed in the final narrative synopsis.

#### Data analysis and synthesis

The characteristics and results of included studies will be summarized using tables, supplemented by a narrative summary that will compare and evaluate the methods used and the principal results among studies. Attempts will be made to structure the narrative summary, where data will be available, around the country of publication, type of systemic infections (e.g. timing of onset, type of systemic infection), type of intervention, type of economic evaluation and methodological features employed among the studies. Methodological characteristics of included studies will be summarized by using a “Characteristics of included studies” table [18].

Further, an attempt will be made to provide a brief commentary on measuring equity dimensions in the included CEA studies. Each CEA will be assessed and discussed in accordance with the criteria recommended by the Global Priority Setting for Health (GPS-Health) to address (i) the disease and the intervention, (ii) the characteristics of social groups and the (iii) protection against the financial and social effects of ill health [40].

Tabulation of available unit cost data will be done. The currency and price year will be reported. Available costs, incremental costs and cost-effectiveness will be converted to 2017 International Dollars value using implicit price deflators for GDP and GDP Purchasing Power Parities as recommended by CCEMG [21, 41].

#### Assessing the quality of economic evidence

Attempts will be made to assess the quality of economic evidence for outcomes related to “important/critical resource use” as recommended by the “Grading of Recommendations Assessment, Development, and Evaluation” (GRADE) approach [42]. This involves quality assessment based on the following five domains: (1) “Risk of bias/Study limitations”, (2) “Consistency of results”, (3) “Directness of evidence”, (4) “Imprecision” and (5) “Publication Bias”. A GRADE evidence profile and summary of findings (SoF) table will be developed using GRADE Pro Software [42].

#### Discussion

Neonatal systemic infections can give rise to immediate and long-term health effects. Additionally, they have significant economic repercussions on the families, health care providers, societies and the nation. Evidence from health economics studies are vital for all stakeholders dealing with neonatal systemic infections to make comprehensive decisions at the individual, societal, regional and national levels. Pediatric economic evaluations have mainly focused on the under-five age group [8–11], and published reviews of economic evidence for the neonatal age group are missing.

To the best of our knowledge, this is the first review which aims to review the economic evidence on neonatal systemic infections in the South Asian context. Additionally, this protocol provides an understanding of the methods used to design and evaluate economic evidence for methodological quality, transparency, and focus on health equity. This review will also highlight existing gaps in research and identify scope for further research.

## Additional files

Additional file 1: PRISMA-P 2015 Checklist. Consists of completed PRISMA-P checklist denoting the list of items included in the protocol against the PRISMA-P checklist and location of the items in the manuscript files. (DOCX 31 kb)

Additional file 2: Planned outcomes of the systematic review, Preliminary search strategy for MEDLINE (Ovid) and Draft data extraction form. Consists of supplementary tables detailing the (1) Planned outcomes and the respective outcome measures of the systematic review, (2) the preliminary search strategy for MEDLINE (via Ovid) and (3) A draft data extraction form displaying the items for which data will be extracted in the systematic review. (DOCX 25 kb)

## Abbreviations

CBA: Cost-Benefit Analysis
CCEMG: Cochrane Campbell Economic Methods Group
CEA: Cost-Effectiveness Analysis
CHEC: Consensus Health Economic Criteria
CHEERS: Consolidated Health Economics Evaluation Reporting Standards
CMA: Cost-Minimization Analysis
CoI: Cost-of-illness
CUA: Cost-Utility Analysis
DALY: Disability Adjusted Life Years
EED: Economic Evaluation Database
GIN: Guidelines International Network
GRADE: Grading of Recommendations Assessment, Development, and Evaluation
HTAi: Health Technology Assessment International
iHEA: International Health Economics Association
ISPOR: International Society of Pharmacoeconomics and Outcomes Research
NHS: National Health Service
NICE: National Institute for Health and Care Excellence
QALY: Quality Adjusted Life Years
QoL: Quality of life
RePEc: Research Papers in Economics
SAARC: South Asian Association for Regional Cooperation
SoF: Summary of Findings
