# Supplementary material for: A protocol for a systematic review of economic evaluation studies conducted on neonatal systemic infections in South Asia
Source: Syst Rev. 2017 Dec 12;6:252. doi: 10.1186/s13643-017-0648-7 (PMC5727883; doi:10.1186/s13643-017-0648-7)
Supplement: Supplementary file 2 — Planned outcomes of the systematic review, Preliminary search strategy for MEDLINE (Ovid) and Draft data extraction form. Consists of supplementary tables detailing the (1) Planned outcomes and the respective outcome measures of the systematic review, (2) the preliminary search strategy for MEDLINE (via Ovid) and (3) A draft data extraction form displaying the items for which data will be extracted in the systematic review. (DOCX 25 kb) [file 13643_2017_648_MOESM2_ESM.docx]

**Additional File 2**

**Planned outcomes of the systematic review, preliminary search strategy for MEDLINE (Ovid) and Draft data extraction form**

**1. Planned outcomes of the systematic review^[[1]](#footnote-1)^**

| Outcome | Outcome measures |
| --- | --- |
| 1. Resource use: Number and length (where available) of all resources used for management of neonatal systemic infection | |
| 1. Direct Medical | Related to healthcare resources utilized for management of neonatal systemic infection.  e.g. Outpatient visits, consultations, hospitalizations (in-patient visits), provider services (physician, specialist, nursing, respiratory therapist, counsellor), drugs, supplies, devices and medical assistive equipment, investigations and procedures (clinical, laboratory, imaging). |
| 1. Direct Non-medical | Related to non-healthcare resources utilized for the management of  neonatal systemic infection  e.g. Services (e.g. administration and housekeeping), overheads, equipment, transportation, meals, accommodation, relocation and moving, clothing, property losses, informal care (e.g. travel and waiting time) |
| 1. Indirect | e.g. Number of hours/days of work lost (by the guardian/parent) as a result of the neonate’s illness |
| 1. Costs: Estimates of costs of resources used to manage neonatal systemic infections. | |
| 1. Cost items | Related to specific resource items used in the management of neonatal systemic infection  e.g. cost of hospital stay, costs of outpatient visits, costs of consultations, costs of meals, costs of informal care, cost of ventilator, cost of imaging, loss of wages, productivity losses, cost of transport, cost of relocation, cost of provider services (e.g. specialist, physician, nursing, housekeeping) |
| 1. Categories of cost | Direct medical  Direct non-medical  Indirect |
| 1. Cost level | Average costs  Average total direct medical  Average total direct non-medical costs |
| 1. Cost-effectiveness | Incremental cost-effectiveness ratio (ICER)  Incremental cost-benefit ratio  Incremental cost per QALY (quality-adjusted life years)  Incremental cost per DALY (disability-adjusted life years)  Net costs |

**2. Preliminary search strategy for MEDLINE (Ovid)^[[2]](#footnote-2)^**

|  | exp Economic Development/ or exp Models, Economic/ or exp Economic Competition/ or exp Inflation, Economic/ or exp Economic Recession/ or exp “fees and charges”/ or exp Budgets/ |
| --- | --- |
|  | exp "costs and cost analysis"/ |
|  | (economic* or pharmacoeconomic* or "economic analysis" or "economic evaluation" or "economic stud*" or "economic modelling" or price* or pricing).mp. |
|  | ("cost benefit" or "cost effective" or "cost analysis" or "cost minimisation" or "cost utility analysis").mp. |
|  | (cost or "cost description" or "cost saving" or "cost shar*" or "cost allocation" or "cost of illness").mp. |
|  | ("deductibles and coinsurance" or (fiscal or funding or financial or finance)).tw. |
|  | “health?care adj cost$”.mp. |
|  | ((low adj cost) or (high adj cost)).mp. |
|  | (cost adj estimate*) or ((cost adj variable*) or (unit adj cost*).mp. |
|  | **(1 or 2 or 3 or 4 or 5 or 6 or 7 or 8 or 9)** |
|  | exp Sepsis/ or exp "Bone Diseases, Infectious"/ or exp "Arthritis, Infectious"/ or exp "Urinary Tract Infections"/ or exp Pneumonia/ or exp Meningitis/ |
|  | (septicaemia or septicaemia or sepsis).mp. |
|  | pneumon*.mp. |
|  | mening*.mp. |
|  | osteomye*.mp. |
|  | (urethritis or cystitis).mp |
|  | (bacteraemia or bacteria* or pyogen*).mp |
|  | ((arthritis adj1 (infect* or septic or bacteria* or pyogen*)) or (arthritis adj2 (infect* or septic or bacteria* or pyogen*)) or (arthritis adj3 (infect* or septic or bacteria* or pyogen*))).mp. |
|  | ((infection adj1 (blood or lung or bone* or systemic or "urinary*" or respirat* or joint or brain or generalised)) or (infection adj2 (blood or lung or bone* or systemic or "urinary*" or respirat* or joint or brain or generalised)) or (infection adj1 (blood or lung or bone* or systemic or "urinary*" or respirat* or joint or brain or generalised)) or (infection adj3 (blood or lung or bone* or systemic or "urinary*" or respirat* or joint or brain or generalised))).mp. |
|  | **(11 or 12 or 13 or 14 or 15 or 16 or 17 or 18 or 19)** |
|  | exp "Infant, Newborn"/ |
|  | infan*.mp. |
|  | (neonate or newborn).mp. |
|  | (baby or babies or toddler).mp. |
|  | (paediatric* or pediatric*).mp. |
|  | **(21 or 22 or 23 or 24 or 25)** |
|  | (Afghanistan* or Bangladesh* or Bhutan* or Maldives or Nepal* or Pakistan* or "Sri Lanka").ti,ab,kw,cp,in |
|  | Afghanistan/ or Bangladesh/ or Bhutan/ or India/ or Nepal/ or Pakistan/ or Sri Lanka/ |
|  | ((India or Indian) not ((“American indian*” or “west indian*”) or (exp Indians, central american/ or exp Indians, south american/ or exp Indians, north american))).ti,ab,kw,cp,in |
|  | **(27 or 28 or 29)** |
|  | **(((10) and (20 and 26) and 30)** |
|  | exp Animals/ NOT exp Humans/ |
|  | **(31 not 32)** |

Note: mp=title, abstract, text word, author keywords, outline heading, caption text, full text

#27 includes country of publication and institution

Note: In the absence of database-specific filters for “Humans”, we will use the following method to restrict studies to ‘human population”

1 [result of topic search]

2 (Animals/ NOT Humans/)

3 1 NOT 2

**3. Draft data extraction form^[[3]](#footnote-3)^**

| Sl.No | Items Category | Sub items | Notes |
| --- | --- | --- | --- |
|  | Source | study ID, review author ID, citation, title of study, contact details of the corresponding author |  |
|  | Eligibility | eligibility decision |  |
|  | Study objective(s)/ research question | |  |
|  | Study methods | study design  type of economic analysis  study setting, total study duration, sample size, sample size calculation, recruitment, selection and allocation methods  perspective: e.g. societal, health care system, service provider (e.g. hospital), health care program, third party (e.g. social insurance) |  |
|  | Data collection methods | Quantitative data: time-motion study, patient self-report, medical records, databases, interview, etc  Unit cost data collection: invoice amount, hospital/clinic/provider price catalogue, national/regional/provincial/hospital/insurer fee schedule, human resources/payroll record, etc  Methods used to define effectiveness and preferences |  |
|  | Participant Characteristics | total number, age in days/ weeks, sex, country, socio-demographics, ethnicity, etc |  |
|  | Disease Characteristics | diagnostic criteria,  classification used [e.g International Classification of Diseases- Ninth Revision (ICD-9)] |  |
|  | Intervention and comparator characteristics | |  |
|  | Costing characteristics | Costing year, Inflation adjustment, Currency unit, Currency conversion rate to USD |  |
|  | Cost components | Consultation, transport, medicine/ drugs, nursing, care facility, transport, consumables, personnel, etc |  |
|  | Time horizon | |  |
|  | Discounting and rate | yes/no  Rate of discounting utilized |  |
|  | Study Outcome | Economic outcome(s) |  |
|  | Ingredients approach | yes/no |  |
|  | Analysis of uncertainty | |  |
|  | Study assumption(s) | |  |
|  | Sensitivity analyses | Yes/ No  Type of sensitivity analysis (e.g. deterministic, stochastic) |  |
|  | Miscellaneous | Key conclusions of study authors  Limitations of the study  Miscellaneous comments from study authors  Funding source  Conflict of interest,  References to other relevant studies |  |
|  | Reviewer comments | Correspondence required  Miscellaneous comments by review authors |  |

1. ## Adapted from sources cited in the “Criteria for considering studies for this review” of the main article

   [↑](#footnote-ref-1)
2. Adapted from sources cited in the “The search strategy” section of the main article [↑](#footnote-ref-2)
3. Adapted from sources cited in the “Data Extraction” section of the main article [↑](#footnote-ref-3)
